# Should we ligate coronary conduits when flow measurements indicate competitive flow? a case report

**DOI:** 10.1186/s13019-025-03529-0

**Published:** 2025-12-02

**Authors:** Konstantinos Papakonstantinou, Panagiotis Lerios, Mihalis Argiriou, Vasilios Patris, Ilias Gissis, John Kokotsakis, Panagiotis Dedeilias, Periklis Tomos

**Affiliations:** 1 Department of Cardiothoracic and Vascular Surgery, Evangelismos General Hospital of Athens, Athens, Greece; 2 “Attikon” University Hospital of Athens, Athens, Greece

**Keywords:** Transit-time flow measurement, Competitive flow, Case report

## Abstract

**Background:**

Competitive flow (CF) in coronary artery bypass grafting (CABG) occurs when graft flow is impaired due to higher competing flow from either the native circulation or another graft and may result in functional graft occlusion. We report a case of functional graft occlusion of a left internal mammary artery (LIMA) due to flow competition with a saphenous vein graft (SVG) and discuss the rationale of ligating the graft responsible for the CF.

**Case presentation:**

The case refers to a 56-year-old man with an isolated subtotal ostial left anterior descending (LAD) lesion who underwent conventional double on-pump CABG with an in situ (LIMA) to the LAD and a centrally anastomosed SVG to the Diagonal branch (D1). Concerning the initial Transit-time flow measurements (TTFM), the SVG had a mean graft flow (MGF) of 49 ml/min, a Pulsatility index (PI) of 1 and a Diastolic flow (DF) of 66%. The LIMA graft had an MGF of 31 ml/minute, a PI of 3.8, a DF of 80%, but a Backward flow (BF) of 8%. As the LIMA BF was quite high, we suspected competitive flow of the graft and thus decided to transiently apply a bulldog clamp to the SVG, to check if the LIMA TTFM would change. Indeed, MGF was 44 ml/minute, the PI was 2.3, DF was 60%, and BF was 0.9%. Despite these results, the operating surgeon did not alter the revascularization plan and the operation was finished. The patient was discharged uneventfully. However, in a 6-month follow-up, the patient was symptomatic and with poor ejection fraction, and the diagnostic work-up revealed the functional occlusion of the LIMA graft with a string sign, and a patent SVG. After Heart Team consultation and patient update, he underwent left main stent implantation.

**Conclusions:**

There should be a great degree of suspicion for CF with high BF values, even when the rest of the parameters measured during TTFM are normal or marginal. Also, the location of the LAD stenosis proximal to the D1 is a risk factor for CF when both arteries are grafted. Changing the revascularization plan based on TTFM is warranted for optimal outcomes.

**Supplementary Information:**

The online version contains supplementary material available at 10.1186/s13019-025-03529-0.

## Background

Backward flow (BF) in coronary artery bypass grafting (CABG) as measured by transit-time flow measurement (TTFM) is affected by native coronary artery stenosis, with proportionate decrease of BF with greater severity of stenosis [1]. This observation is directly related to the native flow being lower than the bypass graft flow. When the opposite happens, “competitive flow” is present.

Di Giammarco et al. explicitly described the term “functional occlusion” of bypass grafts [2]. Low Mean Graft Flow (MGF) along with high Pulsatility Index (PI) and high BF values suggest competitive flow through a patent graft which has a high probability of late failure due to diminished downward runoff.

Herein, we describe a case report of functional occlusion of a left internal mammary artery (LIMA) graft due to competitive flow from a centrally anastomosed saphenous vein graft (SVG). We also discuss the dilemma of ligating the venous graft.

This case report was prepared using the CARE guidelines [3].

## Case presentation

**Fig. 1:**
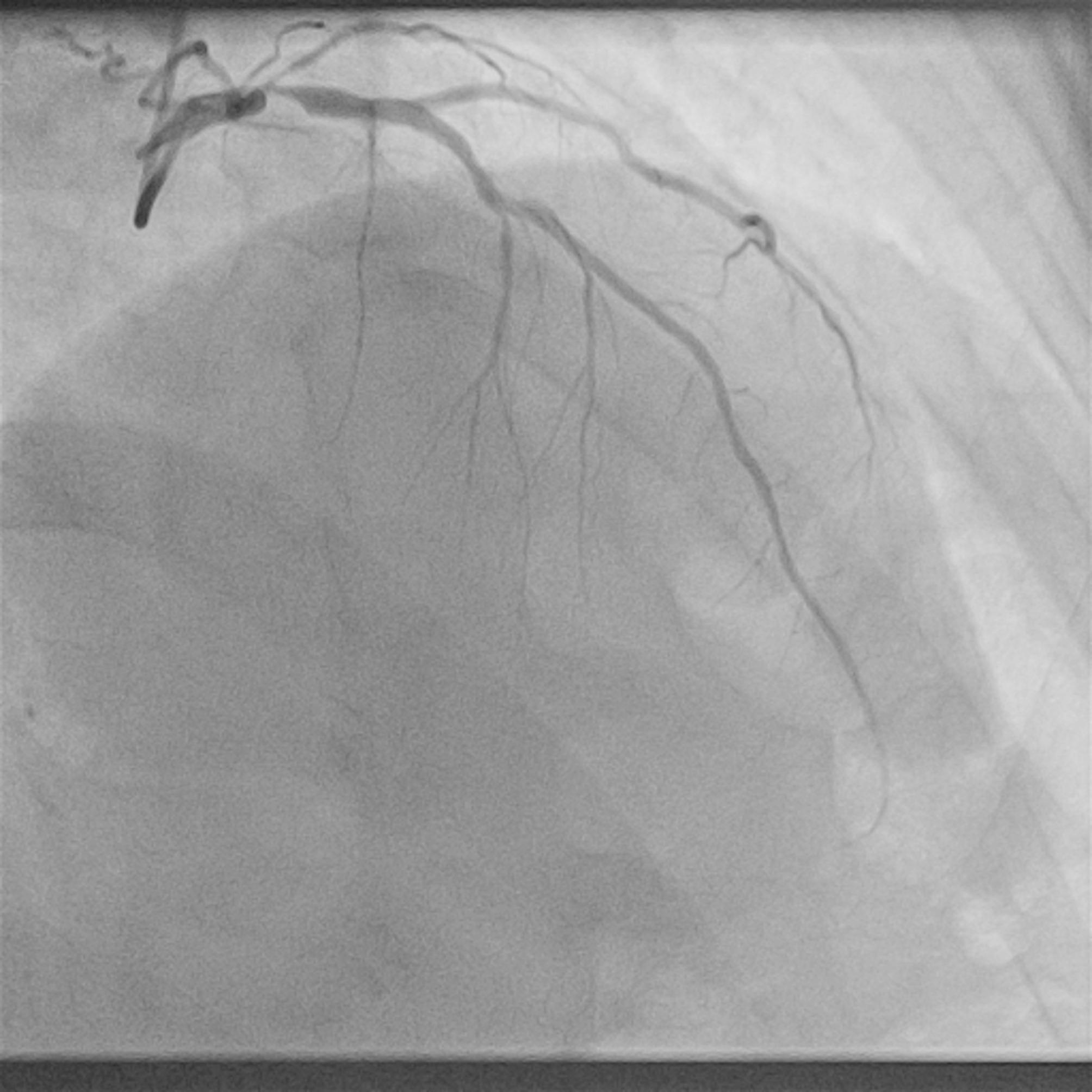
Pre-operative coronary angiography showing the sub-total ostial LAD lesion. LAD: left anterior descending

**Fig. 2:**
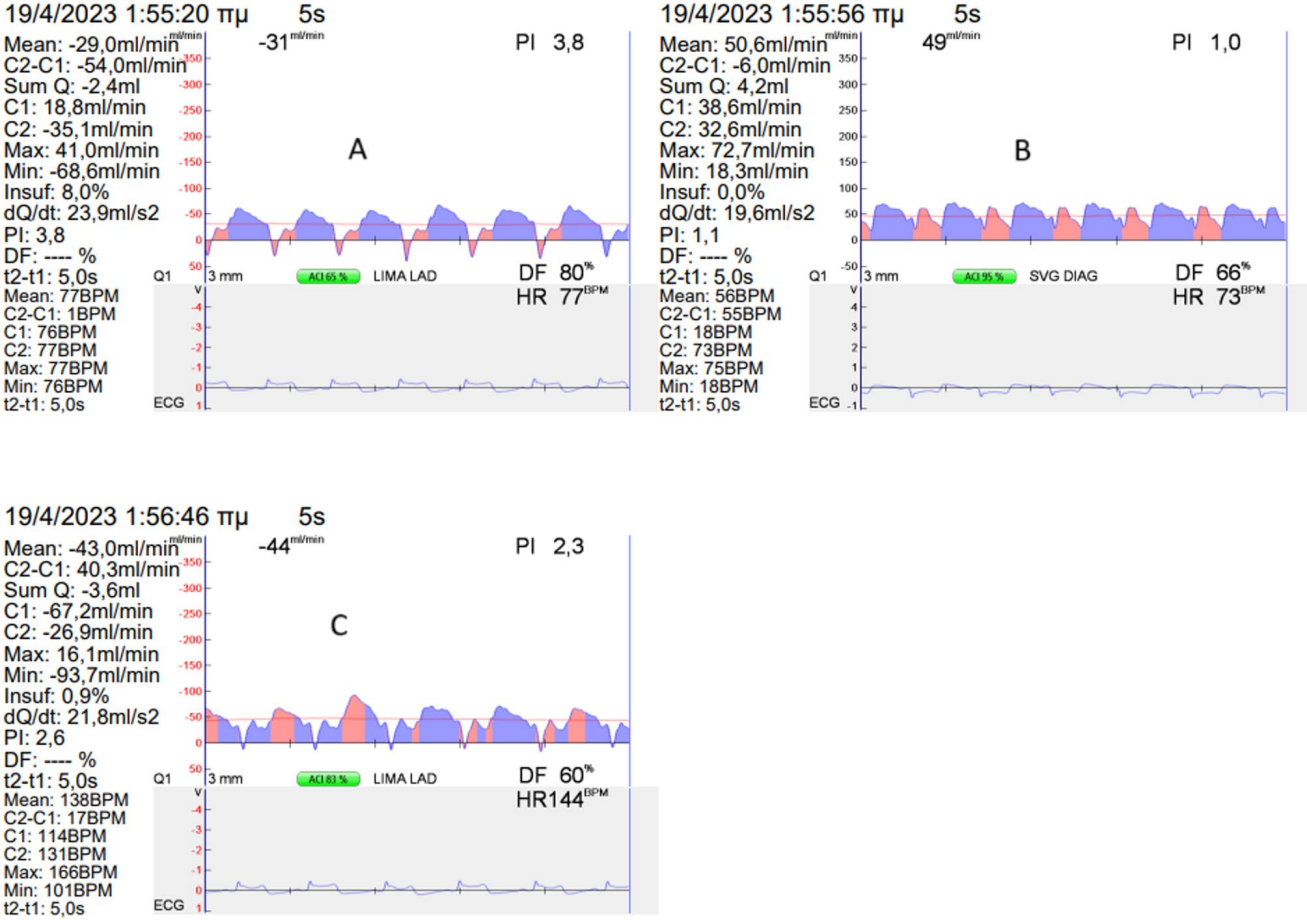
Intraoperative transit-time flow measurements of the bypass grafts. Measurement **A** refers to LIMA graft hemodynamics prior to SVG clamping, Measurement **B** refers to SVG hemodynamics and Measurement **C** refers to improved LIMA graft hemodynamics after SVG clamping. DIAG: diagonal branch, LAD: left anterior descending, LIMA: left internal mammary artery, SVG: saphenous vein graft

A 56-year-old male was referred to the Department of Cardiothoracic Surgery of our hospital for CABG in April 2023. His medical record included hypertension, hyperlipidemia, and past smoking. The patient had a positive stress-echo for anterior wall ischemia and showed a normal ejection fraction. Coronary angiography was carried out, which revealed an isolated subtotal ostial LAD lesion (Fig. 1). The patient underwent conventional on-pump double CABG, with an in-situ LIMA anastomosed to the left anterior descending (LAD) and a SVG anastomosed to the Diagonal Branch (D1). Intraoperative TTFM of the bypass grafts, however, had some important findings (Fig. 2):

- The SVG had an MGF of 49 ml/min, a PI of 1.0, and a DF of 66%.
- The LIMA graft had an MGF of 31 ml/min, a PI of 3.8, and a DF of 80%. However, BF was 8%.

Due to high LIMA BF, we suspected competitive flow of the graft. Thus, we transiently applied a bulldog clamp to the SVG proximally and observed any alterations on the LIMA TTFM measurements in a course of more than seven heartbeats. Indeed:

- LIMA MGF was 44 ml/min, PI was 2.3, DF was 60%, and BF was 0.9%.

The bulldog clamp was retracted, and the operation was completed without any intraoperative cardiovascular events.

The patient’s postoperative course was uneventful, and he was discharged on postoperative day 5.

During routine 6-month follow-up, the patient complained about new-onset exertional dyspnea and was therefore submitted to single-photon emission computed tomography myocardial perfusion imaging. The test was negative for ischemia and showed a myocardial scar of the apex. Therefore, the patient was conservatively treated with guideline-directed medical therapy.

The patient, however, insisted on his symptoms and underwent a stress-echo. Findings included anterior, basal and mid inferoseptal wall stress ischemia, an ejection fraction of 40% and the akinetic scarred apex. Consequently, the patient was referred for coronary angiography. Imaging results were the following:

- The SVG was patent, and the contrast injected in the graft was evident in both the D1 and the LAD. Also, there was a subtotal occlusion of the D1 on the toe of the anastomosis (Supplemental Video 1).
- There was a string sign of the LIMA along with sluggish antegrade flow, signs indicative of functional occlusion of the graft. In addition, there was an opacification of an unligated side branch (Supplemental Video 2).

**Fig. 3:**
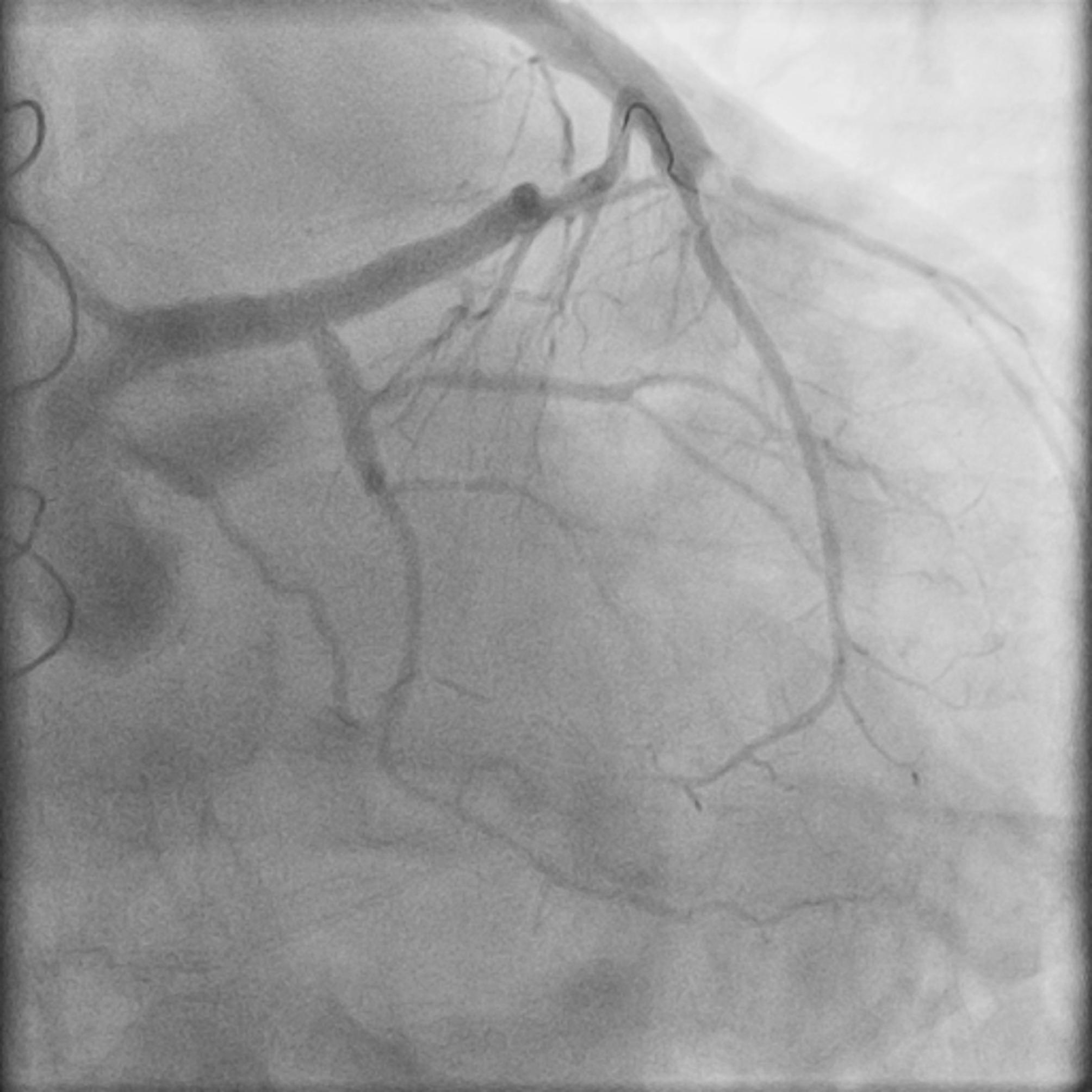
Final angiography after PCI of the left main bifurcation. PCI: percutaneous coronary intervention

After Heart Team consultation and patient update, a shared-decision process was carried out and the patient was submitted to percutaneous coronary intervention (PCI) of the LAD lesion. Due to anatomic factors, the left main stem and the circumflex artery were also stented using the reverse mini-crush technique (Fig. 3). The patient’s course was unremarkable and was discharged home.

## Discussion and conclusions

This case report best describes a dilemma posed during surgical revascularization using TTFM measurements.

Intraoperatively, the LAD was found to be diffusely diseased and of small caliber. A second anastomosis to the D1 branch using a vein graft was considered mandatory by the operating surgeon to enhance the anterior wall revascularization, which was, nevertheless, proven unnecessary, as well as harmful. The location of the LAD stenosis proximal to the D1 is a risk factor for competitive flow when both arteries are grafted, and the obstruction of the toe of the SVG-D1 anastomosis augmented the retrograde flow from the D1 to the LAD [4, 5]. The latter could not have been detected, as the D1 TTFM measurement was considered adequate. Despite this occurrence, however, the question remains as to how to proceed with the patient, and the goal is to preserve LIMA function. Notably, the optimal grafting strategy would be a single LIMA-LAD graft, but since another revascularization plan was followed and competitive flow was indicated, alternatives in this case may include:

- ligating the venous graft.
- LIMA-D1-LAD sequential grafting.

Altering the revascularization plan when competitive flow occurs is warranted to prevent functional graft occlusion. These scenarios may include composite grafting, the right coronary territory without highly significant stenosis, moderate native coronary stenoses in the left system, or sequential grafting [2, 6–10]. For example, a single inflow source in Y-grafting may become dual, with one arm of the Y-graft separated as a free graft [2, 11]. Of course, prevention of these occurrences with a thorough plan is of outmost importance. Special consideration should be given to arterial grafts, since they are more prone to competitive flow than vein grafts due to their self-regulating capacity to adapt to myocardial demands [12].

We considered factors that may have influenced our results. First, TTFM measurements were carried out based on a protocol produced by experts in the field [13]. Although LIMA TTFM cutoffs prior to D1 clamping concerning MGF, PI and DF were not met, a high percentage of BF urged us to reassess LIMA for competitive flow. Since the LAD lesion was quite tight, the only possible reason for competitive flow would be the D1 graft, rather than antegrade LAD flow. Clamping the D1 serves as a functional test, as indicated by the above protocol in cases of competitive flow, and was carried out first to watch how LIMA TTFM measurements would change, but also to check for ECG or wall motion abnormalities, and to assess the patient’s hemodynamic status, as if the SVG was not present. Furthermore, secondary prevention measures, that may have influenced graft patency (guideline-directed medical therapy on discharge, compliance with medical treatment on follow-up, smoking relapse) were also assessed and considered adequate.

There should be a great degree of suspicion for competitive flow with high values of Backward flow, even when the rest of the parameters measured during TTFM are normal or marginal. DiGiammarco et al., in his seminal paper, describes a high risk of functional occlusion at follow-up when a combination of MGF < 15 ml/min, a PI of > 3.0, and BF of > 3.0 is present [2]. In our case, the only pre-clamping measurement that was marginally abnormal was a PI of 3.8, with a general cut-off value of 3.5 for grafts implanted in the left circulation. As a result, caution is advised when interpreting the results of TTFM measurements, and changes in the revascularization plan should be made accordingly to avoid detrimental complications. A greater than arguably normal graft flow may be considered “inadequate” for a specific graft based on the surgeon’s anticipation of a “higher than normal” graft flow.

## Electronic supplementary material

Below is the link to the electronic supplementary material.

Supplementary Material 1

Supplementary Material 2

## Data Availability

No datasets were generated or analysed during the current study.

## Abbreviations

CABG: Coronary artery bypass grafting
TTFM: Transit-time flow measurement
MGF: Mean graft flow
PI: Pulsatility index
BF: Backward flow
DF: Diastolic filling
LIMA: Left internal mammary artery
LAD: Left anterior descending
D1: Diagonal branch
SVG: Saphenous vein graft
